# Retraction: Prevalence and determinants of modern contraceptive utilization among women in the reproductive age group in Edaga-hamus Town, Eastern zone, Tigray region, Ethiopia, June 2017

**DOI:** 10.1371/journal.pone.0275607

**Published:** 2022-09-28

**Authors:** The PLOS ONE Editors

During an internal investigation carried out following publication of [1], PLOS requested documentation in support of the statement in the Methodology section of [1] that ethical clearance for this work was obtained from the Ethical Review Board of Adigrat University, College of Medicine and Health Sciences. The documents provided to PLOS in follow-up discussions did not resolve the pending questions about ethics approval and instead raised additional concerns.

PLOS has been unable to discuss this matter with a representative of Adigrat University; however, it is understood that substantial communication issues have been affecting the region for an extended period.

As we have been unable to clarify whether this work received ethics approval, and in light of concerns identified in our assessment of the provided ethics documents, the *PLOS ONE* Editors retract this article.

TGG, LG, and HNT did not agree with retraction. TGG and LG stand by the article’s findings. DT, MGH, TG, and ZGA could not be reached.

## References

[1] Tukue D, Gebremeskel TG, Gebremariam L, Aregawi B, Hagos MG, Gebremichael T, et al. (2020) Prevalence and determinants of modern contraceptive utilization among women in the reproductive age group in Edaga-hamus Town, Eastern zone, Tigray region, Ethiopia, June 2017. PLoS ONE 15(3): e0227795. doi: 10.1371/journal.pone.0227795 32142517 PMC7059931

